# Congenital Diarrheal Disorders: An Updated Diagnostic Approach

**DOI:** 10.3390/ijms13044168

**Published:** 2012-03-29

**Authors:** Gianluca Terrin, Rossella Tomaiuolo, Annalisa Passariello, Ausilia Elce, Felice Amato, Margherita Di Costanzo, Giuseppe Castaldo, Roberto Berni Canani

**Affiliations:** 1 Department of Gynecology-Obstetrics and Perinatal Medicine, University of Rome “La Sapienza”, Viale del Policlinico 1, Rome 00161, Italy; E-Mail: gianluca.terrin@uniroma1.it; 2 CEINGE-Advanced Biotechnology, Via Comunale Margherita, Naples 80131, Italy; E-Mails: tomaiuolo@dbbm.unina.it (R.T.); elce@dbbm.unina.it (A.E.); amato@ceinge.unina.it (F.A.); castaldo@dbbm.unina.it (G.C.); 3 Department of Biochemistry and Biotechnology, University of Naples “Federico II”, Via Pansini 5, Naples 80131, Italy; 4 Biotechnology Science, University of Naples “Federico II”, Via De Amicis, Naples 80131, Italy; 5 Department of Pediatrics, University of Naples “Federico II”, Via Pansini 5, Naples 80131, Italy; E-Mails: annalisa.passariello@unina.it (A.P.); mara.dicostanzo@live.it (M.D.C.); 6 European Laboratory for the Investigation of Food Induced Diseases, University of Naples “Federico II”, Via Pansini 5, Naples 80131, Italy

**Keywords:** molecular analysis, osmotic diarrhea, secretory diarrhea, defects of digestion, absorption and transport of nutrients and electrolytes, defects of enterocyte differentiation and polarization, defects of enteroendocrine cells differentiation, defects of modulation of intestinal immune response

## Abstract

Congenital diarrheal disorders (CDDs) are a group of inherited enteropathies with a typical onset early in the life. Infants with these disorders have frequently chronic diarrhea of sufficient severity to require parenteral nutrition. For most CDDs the disease-gene is known and molecular analysis may contribute to an unequivocal diagnosis. We review CDDs on the basis of the genetic defect, focusing on the significant contribution of molecular analysis in the complex, multistep diagnostic work-up.

## 1. Introduction

Congenital diarrheal disorders (CDDs) are a group of inherited enteropathies with a typical onset early in the life [1–3]. Most CDDs display similar clinical presentation despite different outcomes. For many of these conditions, severe chronic diarrhea represents the main clinical manifestation, while in others, diarrhea is only a component of a more complex multiorgan or systemic disease. In the vast majority of cases appropriate therapy must be started immediately to prevent dehydration and long term, and sometimes life-threatening, complications [2]. Milder forms of CDDs, with less severe clinical picture that remain undiagnosed until later ages, have been described. The less severe outcome may depend on a milder effect of mutations in the disease gene [1–3]. In most cases of CDDs the disease-gene is known [4]. Thus, molecular analysis has become a major advantage in the difficult diagnostic approach to a patient with suspected CDDs [5]. We recently proposed a CDDs classification in four groups: (i) defects of digestion, absorption and transport of nutrients and electrolytes; (ii) defects of enterocyte differentiation and polarization; (iii) defects of enteroendocrine cell differentiation; (iv) defects of modulation of intestinal immune response [3]. This classification could be adopted as practical starting point for the complex diagnostic approach to patients with CDDs. This article is mainly focused on the updated diagnostic work up for these conditions pointing out the contribution of molecular analysis.

## 2. The Contribution of Epidemiological Data to the Diagnostic Approach

The exact incidence of most CDDs remains to be established, but in some cases data are available (Tables 1–4). The knowledge of the incidence of CDDs in specific countries and ethnic groups could help the physician. Some CDDs are more frequent in ethnic groups where consanguineous marriages are usual, or in some geographic areas due to founder effect [1–3,6]. For example, congenital lactase deficiency (LD) is particularly frequent in Finland [3,4]; lysinuric protein intolerance (LPI) has a higher incidence either in Finland and in Japan due to founder effect, and a single mutation is typically found in each of the two ethnic groups; also in southern Italy there are several affected patients [7]; congenital sucrose-isomaltase deficiency (SID) may affect up to 5% of the populations in Greenland, Alaska and Canada [8]. Similarly, congenital chloride diarrhea (CCD) is sporadic worldwide and a large genetic heterogeneity has been reported in about 150 patients described so far [9–11], while in some ethnics CCD has a higher frequency due to founder effect in Finland, Saudi Arabia, Kuwait and Poland.

## 3. The Initial Steps of Diagnostic Work Up

**Figure 1:**
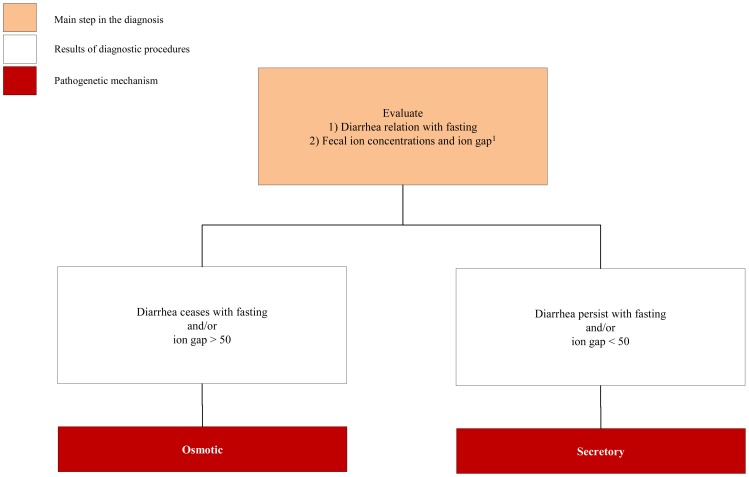
Identification of pathogenetic osmotic or secretory mechanisms leading to diarrhea.

**Figure 2:**
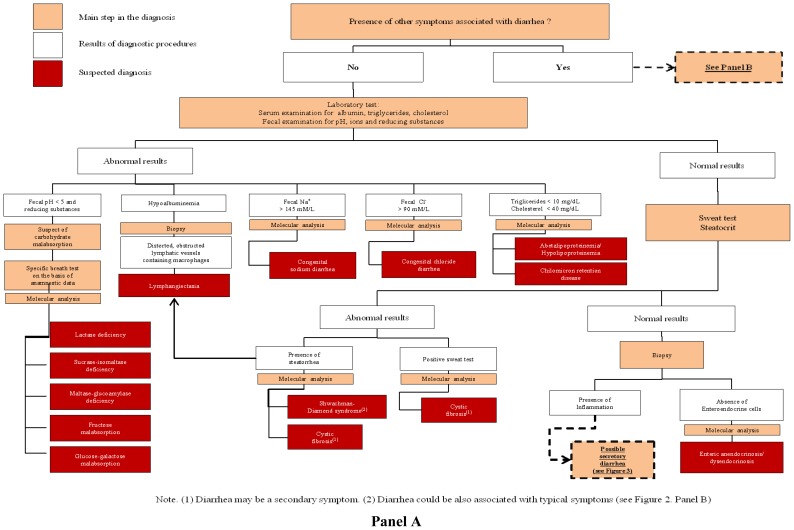

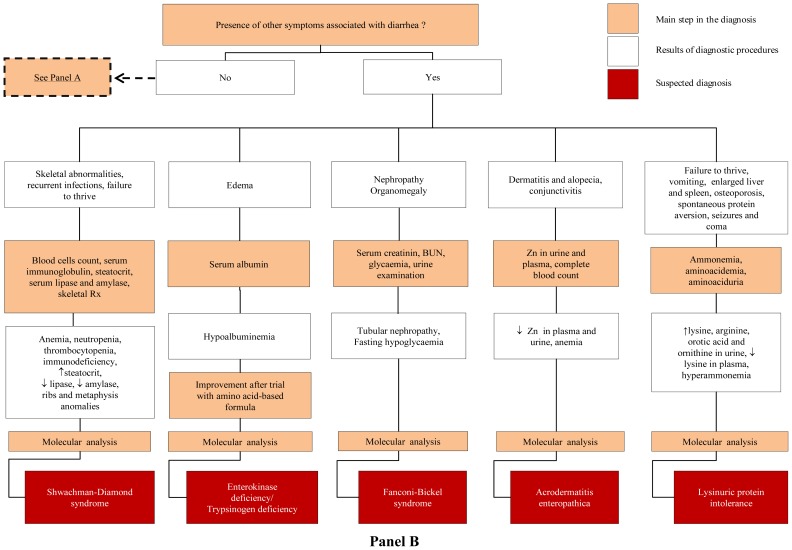
**Panel A.** Diagnostic diagram of congenital diarrheal disorders determined by an osmotic mechanism. **Panel B.** Diagnostic diagram of congenital diarrheal disorders determined by an osmotic mechanism.

**Figure 3:**
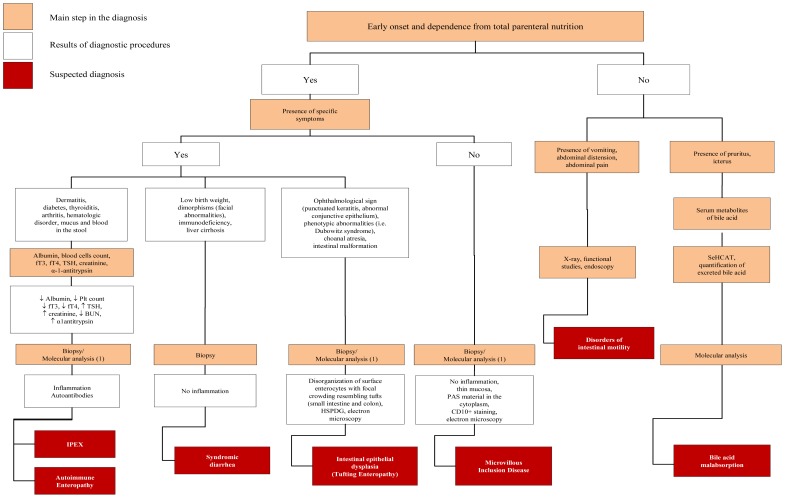
Diagnostic diagram of congenital diarrheal disorders determined by a secretory mechanism.

The diagnostic approach to CDDs is a multistep process that includes the careful evaluation of the anamnesis and clinical data, results of common laboratory and instrumental procedures and molecular analysis (Figures 1–3). Positive familiar history of early onset chronic diarrhea, polyhydramnios and/or dilated bowel loops at ultrasound examination during pregnancy are highly suggestive of CDDs. Frequently CDDs dates to early neonatal period [2,3]. However in the approach to a newborn or infant with suspected CDDs it is important to remember that also at this particular age, infections and food allergy are frequent causes of chronic diarrhea [6], and that these conditions together with malformations of gastrointestinal tract should be considered as primary hypothesis [2,3].

Once suspected, the fundamental step in the diagnostic process of CDDs is the identification of an osmotic or secretory mechanism leading to diarrhea (Figure 1). In osmotic diarrhea unabsorbed luminal substances are responsible for accumulation of fluids in intestinal lumen and diarrhea significantly improves during fasting, whereas in secretory diarrhea fluids are actively secreted in the intestinal lumen and diarrhea continues during fasting [12]. Furthermore, the determination of stool electrolyte concentration and fecal ion gap are important to discriminate the two mechanisms responsible of CDDs (Figure 1).

If ion gap is >50 fecal osmolarity derived from ingested osmotically active nutrient or non measured ion (*i.e.*, Cl^−^, Mg^2+^). In contrast a low osmotic gap (<50) is typically observed in secretory diarrhea. It is also important to measure Cl^−^ concentration in the stool to rule out CCD, characterized by low ion gap due to high Cl^−^ fecal loss (>90 mmol/L) [13]. When an osmotic mechanism is suspected the next step of laboratory investigation includes blood gas, blood glucose, ammonium, albumin, triglycerides and cholesterol, aminoaciduria and the search of reducing substances in the stools, steatocrit and sweat test (Figure 2). Finally, intestinal biopsy with hystologic examination is crucial for the diagnosis of most CDDs, more frequently for secretory forms; even if molecular analysis, when available, could limit invasive procedures (Figures 2 and 3).

### 3.1. Congenital Osmotic Diarrheal Disorders

The main conditions included in this subgroup are characterized by carbohydrate malabsorption with subsequent bacteria fermentation and increased lactic acid concentration in stools [14]. The detection of fecal reducing substances by Clinitest (normal value < 0.5%) may suggest carbohydrate malabsorption. A stool pH between 5 and 6 is indicative but, finally, unreliable screening test for the diagnosis of sugar malabsorption. Breath test and diet trials are used to address clinical suspect. Small bowel disaccharidase activity measurement on intestinal biopsy could be avoided especially when molecular analysis is available (Figure 2). In the absence of carbohydrate malabsorption in a patient with osmotic diarrhea, it is essential to determine if steatorrhea is present. Although diarrhea alone may be responsible for an increase fat excretion, and generalized malabsorptive diseases may induce steatorrhea, greater rates of fat malabsorption can be explained only by one or more defects in fat digestion and absorption [2,3]. Fat malabsorption can be divided into three broad categories: intraluminal maldigestion (pancreatic insufficiency), mucosal malabsorption, and post-mucosal malabsorption related to lymphatic obstruction [15,16].

Low triglycerides and cholesterol serum levels are typically observed in children with a- or hypo-betalipoproteinemia (ALP/HLP). Biopsy is useful to confirm diagnosis of ALP/HLP. However, molecular diagnosis may be used at this point of diagnostic work up to avoid invasive procedures. Bile salts are particularly important in lipids digestion and absorption. However, in primary bile acid malabsorption (PBAM) diarrhea is generally secretory in nature (persist while fasting) and is exacerbated by the addition of dietary fats (see also Figure 3). Among osmothic CDDs, the onset of symptoms after ingestion of specific food or the presence of extraintestinal symptoms could help the diagnosis. Fructose malabsorption should be suspected when consumption of fruit juices that contain a high proportion of fructose to glucose or an excessive amount of the non-absorbable carbohydrate sorbitol are associated with diarrhea and abdominal pain [17]. Disorders of protein digestion are unusual but should be considered when evaluating a patient with suspected CDDs. The best characterized disorder of specific protein malabsorption is the enterokinase deficiency (EKD) [18]. Individuals with EKD present diarrhea and hypoproteinemic edema when on diet with intact proteins, while symptoms resolve on an amino acids-based diet. The presence of diarrhea associated with tubular nephropathy, hepatomegaly and abnormal glycogen accumulation, and fasting hypoglycemia suggest the presence of Fanconi-Bickel Syndrome (FBS) [19]. The vesciculus bullous dermatitis, located on the hands, feet, perirectal and oral region, and alopecia are a consequence of Zn^2+^ deficiency that could be due to excessive fecal losses of Zn^2+^ observed in acrodermatitis enteropathica (ADE) [20]. Severe Zn^2+^ deficiency can present also with anorexia, failure to thrive, immunodeficiency and neurologic features (mental lethargy and neurosensory abnormalities) associated with chronic diarrhea. A particular form of osmotic diarrhea genetically determined is the enteric anendocrinosis (or congenital malabsorptive diarrhea, CMD) [21]. The salient pathological fact is that the mucosa of the small intestine is essentially normal, except for the absence of enteroendocrine cells and of the enteric hormones. The number of patients described so far is too small to draw reliable conclusions about the typical clinical picture of CMD. However, the diarrhea in this condition is undoubtedly of osmotic nature. While water is well tolerated, glucose-based oral rehydration solution leads to diarrhea and the patients continue to experience diarrhea while on carbohydrate-free cow’s milk or amino-acids based formulas. These infants may be optimally managed with life-long parenteral nutrition and limited enteral nutrition [21].

### 3.2. Congenital Secretory Diarrheal Disorders

In the approach to the infant presenting with early onset secretive chronic diarrhea intestinal biopsy is generally considered useful to identify the major forms of CDDs included in this subgroup (*i.e.*, enterocytes differentiation and polarization and modulation of intestinal immune response), but also in these cases molecular diagnosis may help to limit invasive approaches (Figure 3). When villous atrophy is not accompanied by an inflammatory infiltrate, defect of enterocyte differentiation and polarization including microvillous inclusion disease (MVID) and congenital tufting enteropathy (CTE) are plausible [22]. For MVID and CTE, pregnancy and delivery are uneventful, and polyhydramnios is unusual. In these conditions severe diarrhea occurs within the first weeks of life and rapidly requires total parenteral nutrition. Several cases of CTE have been reported as being associated with phenotypic abnormalities including dysmorphic facial features, choanal atresia, esophageal atresia, and keratitis. Molecular diagnosis may be an option to avoid radiological exposure [2,3]. Immune dysfunction, polyendocrinopathy X-linked syndrome (IPEX) appears as a secretory diarrhea of inflammatory nature associated with dermatitis, diabetes mellitus, thyroiditis, and hematologic disorders [23–25]. Most patients develop a protein-loosing enteropathy with α-1-antitrypsin loss in stools, and hypoalbuminemia [2]. Diarrhea often starts within 3 months of life; however a later onset has occasionally been described [3].

## 4. Genetic Basis of CDDs

Most CDDs are transmitted as an autosomal recessive trait even if with some exceptions. Below, we report the main genetic characteristics of the 4 groups of CDDs.

### 4.1. Defects of Digestion, Absorption and Transport of Nutrients and Electrolytes

This group can be further divided in various subgroups on the basis of the specific gene defect. The first subgroup includes the deficiency of brush border enzymes, and in particular congenital LD due to mutations within the gene encoding the protein with either lactase and phlorizin hydrolase activities [26]; congenital SID, due to mutations in the gene encoding the protein with both sucrase and isomaltase activity [8], and congenital maltase-glucoamylase deficiency (MGD), putatively due to the maltase-glucoamylase (MGAM) gene defect [8], even if some patients with the disease do not bear pathogenetic mutations in such gene. In fact, cases in which MGD was associated to the deficiency of other brush border enzymes were described, and it has been postulated that a pleiotropic regulator factor of such genes may be impaired in these patients [27].

A large subgroup derives from mutations of genes encoding members of the super-family of solute carriers (SLC). These genes are structurally related and originated by mechanisms of duplication, but despite the homology of the gene sequence and the similarity of the protein architecture, the clinical picture and the outcome of these CDDs is heterogeneous. Most proteins encoded by SLC genes are expressed at intestinal level, thus the disease typically appears with diarrhea and selective malabsorption. In other cases the carrier is expressed also in other organs (*i.e.*, the aminoacid transporter involved in LPI is expressed also in other tissues) and thus the disease could involves other organs or be systemic [7]. For some CDDs disease-genes are still unknown or more genes may be involved. For example, the disease gene for fructose malabsorption is still undefined [8,17,28]. There remains insufficient evidence that glucose transporter (GLUT) 5 represents the main fructose facilitative carrier in the intestine [17,28]. Recent studies have identified GLUT7 as a high affinity glucose and fructose carrier on the brush border membrane of the enterocyte located in the distal intestine. Other studies suggest also a role of GLUT2 in the absorption of large amount of fructose across the enterocyte of the rats. In particular, the disease-gene for congenital sodium diarrhea (CSD) is unknown [3], and a study based on the candidate gene approach failed to identify the responsible among the 6 known isoforms of sodium-proton exchangers (NHE) 1 to 6. However, in addition to classic CSD characterized by perinatal onset of severe diarrhea there is a syndromic form of the disease. The disease gene of the syndromic CSD is serine protease inhibitor (SPINT) 2 [29], which encodes a serine-protease inhibitor, recently identified using genome-wide single nucleotide polymorphism (SNP) linkage analysis in a large family. Mutations of the gene were identified in all other four syndromic patients studied. On the contrary, no mutations were identified in patients bearing the classic form of the disease [29,30]. A third subgroup involves genes encoding specific enzymes like the congenital absence of pancreatic lipase (APL) [31], that is a rare disease associated to malabsorption, steatorrhea and severe diarrhea. Also included in this group are hereditary pancreatitis (HP) [32]. However, it could be important to underline that some cases of chronic hereditary pancreatitis derive from mutations in the cystic fibrosis transmembrane regulator (CFTR) gene [33].

A fourth subgroup of rare and congenital disorders of lipoprotein metabolism can appear with chronic diarrhea. Familial ALP is due to mutations in microsomal triglyceride transfer protein (MTTP) gene which encodes the MTT protein involved in the synthesis of beta lipoproteins [34,35]. Familial HLP is a relatively mild disease that may appear with chronic diarrhea and fatty liver [36]. It depends on co-dominant mutations in the ApoB gene, but in some cases a linkage to chromosome 3 (3p21) was found [37].

The last subgroup includes CDDs deriving from mutations of genes encoding ribosomial proteins. The SDS is a systemic disease due to mutations in the SBDS gene that encodes a protein whose function is still largely obscure [38,39]. Most of these mutations cause exchange of genetic material (gene conversion) between SBDS and a close pseudogene.

### 4.2. Defect of Enterocyte Differentiation and Polarization

CDDs due to defects of enterocyte differentiation and polarization include three rare and severe forms. Congenital microvillous inclusion disease (MVID, DIAR2) is one of the most frequent cause of CDDs in neonates [40]. The disease is due to a loss of function of Myosin5B, a protein involved in the recycling of endosomes, encoded by the MYO5B gene. Molecular analysis can be performed, thus reducing the need of invasive approaches and electronic microscopy [4,40], but some MVID patients have been described with one or none mutations in such gene [4,40]. Congenital tufting enteropathy is a rare, early onset (first days after birth), diarrheal disease [41], due to mutations in the gene encoding the epithelial cellular adhesion molecule (EpCAM). The reduced activity of such protein causes the lack of lateral interactions and desmosomal alterations typical of CTE. Also in such disease, the availability of molecular analysis reduced the need of invasive approaches and of electronic microscopy. Finally, the Tricho-Hepato-Enteric (THE) syndrome, is a rare and poorly tractable chronic diarrhea associated to facial dysmorphism and immunological alterations [42]. In addition to this, for such diseases, molecular analysis will help to obtain unambiguous and rapid diagnosis, thus reducing the need for invasive approaches (Figure 2).

### 4.3. Defects of Enteroendocrine Cells Differentiation

This group includes CMD (DIAR4) and proprotein convertase 1/3 deficiency (PCD). The CMD is very uncommon [43]; it appears with a very severe diarrhea since the first days of life and is characterized by the dysgenesis of enteroendocrine cells (the first name of the disease was enteric anendocrinosis). In some CMD patients molecular analysis revealed mutations in the NEUROG 3 gene that encodes neurogenin 3, a transcriptional factor strongly expressed in endocrine cells and necessary for the development of such cells at intestinal and pancreatic level. CMD patients may develop early onset diabetes [44,45]. Going to PCD, the few cases reported so far had a different age at onset and severity of the disease, which usually appears with small intestine dysfunction and a variable polyendocrinopathy. The disease is due to mutations in the proprotein convertase 1/3 (PCD) gene that encodes a hormone processing enzyme [46].

### 4.4. Defects of Modulation of Intestinal Immune Response

Defects of the modulation of the intestinal immune response includes some well codified entities like IPEX and APS1, and the not well defined IPEX-like syndrome. The IPEX syndrome, a rare X-linked disease, bears to the group of autoimmune polyglandular syndromes and is also known with a long series of acronyms [47,48]. The disease gene (*i.e.*, FOXP3) encodes a protein involved in embryonic development. Molecular analysis contributes to diagnosis, and has been used also for prenatal diagnosis [49], but about one third of symptomatic patients are negative to molecular analysis (they are now indicated as IPEX-like syndrome, see below). APS1 is another of the three codified autoimmune polyglandular syndromes with an autosomal recessive inheritance (even if a family with a dominant transmission was described) [47]. APS1 may be easily diagnosed searching for mutations in the autoimmune regulator (AIRE) gene encoding an autoimmune regulator (AIRE) protein [50,51]. To be noted that quite all affected patients have anti-interferon antibodies in serum. Finally, the IPEX-like syndrome (not X-linked despite the name) is a not well-defined disease, which includes patients with IPEX symptoms with no mutations in FOXP3 gene. In one of these patients a deficiency of CD25 due to a recessive mutation in the interleukin-2 (IL-2) receptor subunit CD25 was found [52].

## 5. Molecular Diagnosis

In the majority of cases genes responsible for CDDs are not particularly large. This permit to use scanning techniques like the gene sequencing for molecular analysis, in order to reduce diagnostic errors. Furthermore, some CDDs in specific ethnic groups are caused by a single mutation due to the founder effect, while the same diseases in other ethnic groups may be due to myriad of different mutations. Among all the diseases listed in the Tables 1–4 only in a few cases molecular diagnosis cannot be routinely performed. They include: (i) congenital MGD, because no mutations have been identified so far in the putative disease gene MGAM in affected patients, suggesting that the disease may depend on other regulatory genes [27]; (ii) FM, in which more genes encoding fructose carriers may be involved [17]; (iii) CSD, in which only syndromic forms have mutations in the SPINT2 gene [38]; (iv) IPEX-like syndrome, for which the disease gene is still unknown even if a single patient had a mutation in the CD25 encoding gene [52]. In some CDDs like GGM, IPEX, CF [49,53] molecular analysis has been used for prenatal diagnosis. Given the severe outcome of most CDDs, and the availability of efficient techniques for gene scanning analysis, the request for prenatal diagnosis will increase in the next future. It is important that prenatal diagnosis would be associated to a careful multidisciplinary counseling to the families [54].

## 6. Conclusions

During the last decade we have observed an impressive improvement in the field of CDDs, in terms of pathogenesis knowledge and in the field of diagnostic approaches. Molecular analysis has changed the diagnostic scenario in CDDs, and lead to reduction of invasive and expensive procedures (Figures 2 and 3). However, some critical points warrant a comment: (i) molecular analysis would be based on scanning procedures [55], including the search for large gene deletions [56], using adequate quality control programs [57] and well trained technologists; (ii) the laboratory must be able to perform functional analysis of novel mutations to demonstrate their pathogenic role; (iii) the negative result of molecular analysis does not exclude the disease, because mutations may involve non-coding, regulatory areas; high throughput sequencing could help to perform extensive analyses [55]; however, also if the mutation is not known, carrier and prenatal diagnosis may be performed using linkage analysis [58]; (iv) some CDDs are very rare; it is necessary that laboratories offer molecular diagnosis also for such “orphan” diseases. All these considerations permit to conclude that a few number of reference laboratories involved in molecular diagnostics for CDDs should be organized in international networks. However, given the number of CDDs, the complexity of genotype-phenotype correlations and the need of multidisciplinary counseling to the families, a strict collaboration between physicians and molecular laboratories is mandatory in this field.

## Figures and Tables

**Table 1:** Inheritance, epidemiology and main pathological mechanisms of the defects of digestion, absorption and transport of nutrients and electrolytes.

Disease	OMIM number	Transmission and incidence	Mechanism
**Genes encoding brush-border enzymes**			

Congenital lactase deficiency (LD)	223000	AR, 1:60.000 in Finland; lower in other ethnic groups	Osmotic
Congenital sucrase-isomaltase deficiency (SID)	222900	AR, 1:5.000; higher incidence in Greenland, Alaska and Canada	Osmotic

Congenital maltase-glucomaylase deficiency (MGD)	--	Few cases described	Osmotic
Enterokinase deficiency (EKD)	226200	AR	Osmotic

**Genes encoding membrane carriers**			

Glucose-galactose malabsorption (GGM)	606824	AR, few hundred cases described	Osmotic
Fructose malabsorption (FM)	138230	-	Osmotic
Fanconi-Bickel syndrome (FBS)	227810	AR	Osmotic
Acrodermatitis enteropathica (ADE)	201100	AR, 1:500.000	Osmotic
Congenital chloride diarrhea (CCD, DIAR 1)	214700	AR, sporadic; frequent in some ethnies	Osmotic
Congenital sodium diarrhea (CSD, DIAR 3)	270420	AR	Osmotic
Lysinuric protein intolerance (LPI)	222700	AR, about 1:60.000 in Finland and in Japan; rare in other ethnic groups	Osmotic
Primary bile acid malabsorption (PBAM)	613291	AR	Secretory
Cystic fibrosis (CF)	219700	AR, 1:2.500	Osmotic

**Genes encoding pancreatic enzymes and pancreatic ions transporters**			

Hereditary pancreatitis (HP)	167800	AD	Osmotic
Congenital absence of pancreatic lipase (APL)	246600	--	Osmotic

**Genes encoding proteins of lipoprotein metabolism**			

Abetalipoproteinemia (ALP)	200100	AR, about 100 cases described; higher frequency among Ashkenazi	Osmotic
Hypobetalipoproteinemia (HLP)	107730	Autosomal co-dominant	Osmotic
Chilomicron retention disease (CRD)	246700	AR, about 40 cases described	Osmotic

**Genes encoding ribosomial proteins**			

Shwachman-Diamond syndrome (SDS)	260400	AR 1:10–200.000	Osmotic

**Table 2:** Inheritance, epidemiology and main pathological mechanisms of the defects of enterocyte differentiation and polarization.

Disease	OMIM number	Transmission and incidence	Mechanism
Microvillous inclusion disease (MVID, DIAR 2)	251850	AR; rare; higher frequency among Navajo	Secretory
Congenital tufting enteropathy (CTE, DIAR 5)	613217	AR; 1:50–100.000; higher frequency among Arabians	Secretory
Tricho-Hepato-Enteric syndrome (THE)	222470	AR, 1:400.000	Secretory

**Table 3:** Inheritance, epidemiology and pathological mechanisms of the defects of enteroendocrine cells differentiation.

Disease	OMIM number	Transmission and incidence	Mechanism
Congenital malabsorptive diarrhea (CMD, DIAR 4)	610370	AR; few cases described	Osmotic
Proprotein convertase 1/3 deficiency (PCD)	600955	AR	Osmotic

**Table 4:** Inheritance, epidemiology and main pathological mechanisms of the defects of modulation of intestinal immune response.

Disease	OMIM number	Transmission and incidence	Mechanism
Autoimmune polyglandular syndrome type 1 (APS1)	240300	AR; AD (1 family)	Secretory
Immune dysfunction, polyendocrinopathy, X-linked (IPEX)	601410	X linked (autosomal cases described), very rare	Secretory
IPEX-like syndrome	--	not X-linked	Secretory

## Abbreviations

ADE: acrodermatitis enteropathica
AIRE: autoimmune regulator
ALP: abetalipoprotenemia
APL: absence of pancreatic lipase
APS1: autoimmune polyglandular syndrome type 1
CCD: congenital chloride diarrhea
CDD: congenital diarrheal disorders
CF: cystic fibrosis
CFTR: cystic fibrosis transmembrane regulator
CMD: congenital malabsorptive diarrhea
CRD: chilomicron retention disease
CSD: congenital sodium diarrhea
CTE: congenital tufting enteropathy
EpCAM: epithelial cellular adhesion molecule
EKD: enterokinase deficiency
FBS: Fanconi-Bickel syndrome
FM: fructose malabsorption
GGM: glucose-galactose malabsorption
GLUT: glucose transporter
HLP: hypobetalipoproteinemia
HP: hereditary pancreatitis
IPEX: immune dysfunction, polyendocrinopathy, X-linked
LD: lactase deficiency
LPI: lysinuric protein intolerance
MTTP: microsomal triglyceride transfer protein
MVID: microvillous inclusion disease
MGD: maltase-glucoamylase deficiency
MGAM: maltase-glucoamylase (gene and protein)
NHE: sodium-proton exchanger
PBAM: primary bile acid malabsorption
PCD: proprotein convertase 1/3 deficiency
SDS: Shwachman-Diamond syndrome
SID: sucrase-isomaltase deficiency
SLC: solute carrier
SNP: single nucleotide polymorphism
SPINT: serine protease inhibitor
THE: Tricho-Hepato-Enteric syndrome
